# Multi-platform optical remote sensing dataset for target detection

**DOI:** 10.1016/j.dib.2020.106362

**Published:** 2020-10-01

**Authors:** Sudhanshu Shekhar Jha, Manohar Kumar, Rama Rao Nidamanuri

**Affiliations:** Department of Earth and Space Sciences, Indian Institute of Space Science and Technology, Valiamala, Thiruvananthapuram, Kerala, India

**Keywords:** Target detection, Multi-platform remote sensing, Engineered material detection, Sub-pixel material detection, AVIRIS-NG, Terrestrial hyperspectral imaging, Field reflectance spectroscopy

## Abstract

Target detection in remote sensing has vital applications in mineral mapping, law enforcement, precision agriculture, strategic surveillance, etc. We present the acquisition of a first-of-its-kind high-resolution multi-platform (ground, airborne, and space-borne) remote sensing-based benchmark dataset for target detection studies. The dataset includes imagery acquired from terrestrial hyperspectral imager (THI), airborne hyperspectral sensor (AVIRIS-NG), and space-borne multi-spectral (Sentinel-2) sensor on 20th March 2018. Five engineered targets of different materials and colours were placed on different surface backgrounds. Besides, in-situ reflectance spectra of the targets were also acquired using a spectroradiometer for serving as a spectral reference source. The airborne and space-borne imagery were processed to remove un-calibrated/noisy bands and were atmospherically corrected using a radiative transfer method based Fast Line-of-sight Atmospheric Analysis of Spectral Hypercubes (FLAASH) model. The in-situ target reflectance spectra were resampled to spectrally match with airborne and space-borne imagery. Further, a target region of interest (ROI) was designated for each of the targets in both airborne and space-borne imagery using the known ground position of targets using a GPS device. This article provides a ground to space integrated target detection dataset, including ground positions ROI of the targets, point, and pixel-based in-situ target reference spectra, and the processed airborne and space-borne imagery to make the dataset ready for use. The data acquired in this experiment is an attempt to assess the potential of engineered material target detection in a multi-scale multi-platform view setup. The dataset is a valuable resource for testing and validation of target detection algorithms from various strategic and civilian application perspectives of remote sensing.

## Specifications Table

**Table:** 

Subject	Engineering (General)
Specific subject area	Remote Sensing, Target detection, Hyperspectral/multi-spectral image analysis
Type of data	Comma Separated Value Spectral Library Image (raster) Region-Of-Interest (ROI)
How data were acquired	In-situ point target reflectance spectra were collected using field spectroradiometer (Spectra Vista Corporation, HR-1024i, USA). In-situ pixel-based target reflectance spectra were collected using push-broom terrestrial hyperspectral imager (Headwall Photonics Inc., USA). The ground position of the targets was recorded using a GPS device. The airborne hyperspectral imagery was acquired using AVIRIS-NG sensor and the space-borne multi-spectral imagery was acquired from the Sentinel-2 sensor.
Data format	Raw/Processed Target detection imagery are in ENVI hdr file format All the processed target reference spectra are provided in ASCII (.txt) and ENVI .sli format. Raw field SVC spectra in .sig file format. Target ground locations are provided in .roi, .shp and ascii (.txt) file format
Parameters for data collection	The targets were acquired by airborne and space-borne platforms in cloud-free atmospheric conditions. The detection imagery was acquired in nearly nadir sensor geometry. The targets were placed in on natural landscape with different background conditions. The targets were positioned in an urban landscape setting.
Description of data collection	Five targets of different materials, each of size 10 m x 10 m were placed in an open environment with vegetation and bright soil backgrounds. The targets were imaged simultaneously by an airborne hyperspectral sensor (AVIRIS-NG) and space-borne multi-spectral sensor (Sentinel-2). The in-situ reflectance spectra of the targets were acquired using a spectroradiometer. Imaging-based in-situ target spectra were collected using a close-range terrestrial hyperspectral imager.
Data source location	City/Town/Region: Gudalur, Tamil Nadu Country: India Below is the description of the target name and its central lat/long GPS coordinates information - T1 – 11.502843 N / 76.494965 E T2 – 11.503132 N / 76.494967 E T3 – 11.502441 N / 76.495660 E T4 – 11.507789 N / 76.489491 E T5 – 11.507608 N / 76.489490 E
Data accessibility	Repository name: Mendeley Data Data identification number: http://dx.doi.org/10.17632/5ph8ms8p5n.2 Direct URL to data: https://data.mendeley.com/datasets/5ph8ms8p5n/2
Related research article	Jha, S.S. and Nidamanuri, R.R., 2020. Gudalur Spectral Target Detection (GST-D): ANew Benchmark Dataset and Engineered Material Target Detection in Multi-Platform Remote Sensing Data. *Remote Sensing*, *12*(13), p.2145. DOI: https://doi.org/10.3390/rs12132145

## Value of the Data

 

• Remote sensing-based target detection, especially using hyperspectral imaging, has evolving various strategic and civilian plications. The existence of a reference dataset for research is scarce, and datasets from multiple platforms are not available so far.
• The multi-platform benchmark dataset presented will be valuable for researchers in remote sensing, artificial intelligence, military surveillance for rigorous validation of the detection algorithms in a holistic environment.
• The dataset can be used to gain insight into how the performance of the spectral target detector varies as a function of sensor and platform. This would help to design detection algorithms invariant to these external factors.
• The global research community working on developing sophisticated spectral detection algorithms can use this data to validate the results, as the dataset contains targets in a realistic complex environment.

## 1 Data Description

**Fig. 1:**
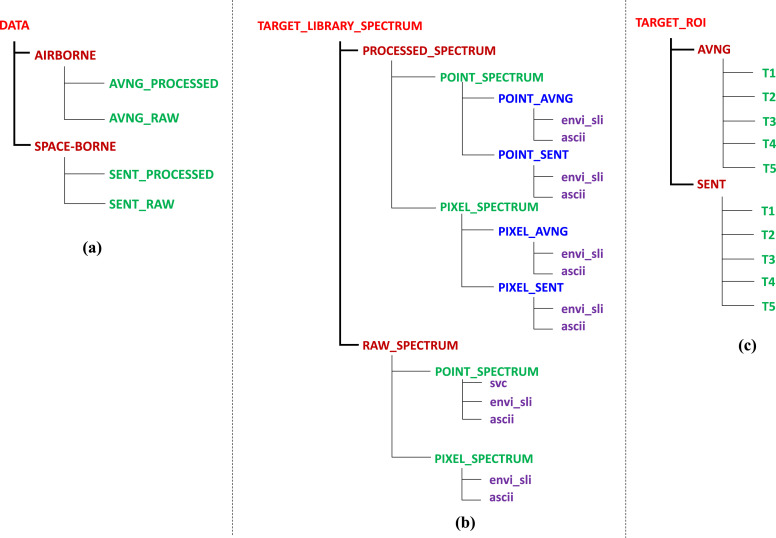
The folder organization of the experimental dataset comprising of (a) raw and processed detection imagery dataset, (b) the field reference raw and processed spectra, and (c) ground positions of the targets.

The hierarchical structure of the dataset is shown in Fig. 1. The data is provided in a zipped archive with the top folder named as Data_in_brief_dataset. The folder Data_in_brief_dataset is organized into three different subfolders – DATA, TARGET_LIBRARY_SPECTRUM, and TARGET_ROI. The DATA folder is further divided into the AIRBORNE and SPACE-BORNE subfolders, which contains the processed and raw subfiles containing respective imagery files provided in ENVI .hdr file format. The ENVI .hdr file contains the metadata information of the associated raster file. The AIRBORNE folder contains two folders: AVNG_PROCESSED and AVNG_RAW. The folder AVNG_PROCESSED contains the processed AVIRIS-NG imagery file AVNG_REFL, and ENVI header file AVNG_REFL.hdr, while the folder AVNG_RAW contains the imagery file AVNG_RAW and ENVI header file AVNG_RAW.hdr. The SPACE-BORNE folder contains two folders: SENT_PROCESSED and SENT_RAW. The folder SENT_PROCESSED contains the processed Sentinel-2 imagery file SENT_REFL, and ENVI header file SENT_REFL.hdr, while the folder SENT_RAW contains the imagery file SENT_RAW and ENVI header file SENT_RAW.hdr. The raw imagery is the radiance version of the data (unit - *μ*W/sr/nm/cm^2^), and the processed imagery has the reflectance version of the data, scaled to 0–10,000. In the case of airborne data (AVIRIS-NG sensor) (Fig. 1(a)), raw imagery (AVNG_RAW) contains 425 bands, whereas processed imagery (AVNG_REFL) contains 370 bands after removing bands sensitive to noise and water vapour absorption regions. Both the raw and processed imagery data bands are organized in interleaved by line (BIL) format having a spatial resolution of 4 m and a spatial extent of 436 × 1171 pixels georeferenced in World Geodetic System (WGS)−84 datum. The space-borne imagery (Sentinel-2) contains ten bands in both the processed (SENT_REFL) and raw (SENT_RAW) imagery. The Sentinel-2 bands are organized in band-sequential (BSQ) format with a spatial resolution of 10 m having a spatial extent of 176 × 470 pixels georeferenced in WGS-84 datum.

The ground-based in-situ target material library spectrum is organized, as shown in Fig. 1(b). The target naming and their description can be found in the section Targets discussed below. The file TARGET_LIBRARY_SPECTRUM contains two subfiles- PROCESSED_SPECTRUM and RAW_SPECTRUM, which includes the processed (resampled to the respective sensor) and raw target material spectrum. The in-situ reference is collected in two modes – point and pixel. The raw target spectra (point and pixel) are provided in the subfolders - POINT_SPECTRUM and PIXEL_SPECTRUM, having a value in the range 0–1 and wavelength units as nanometer(*nm*) and micrometer(*μm*) for pixel and point target spectra, respectively. The PROCESSED_SPECTRUM folder contains two subfolders POINT_SPECTRUM and PIXEL_SPECTRUM, including the target spectra for each sensor (in subfolders POINT_AVNG, POINT_SENT, PIXEL_AVNG, and PIXEL_SENT) convolved to the respective wavelength of the sensors. The folders: envi_sli and ascii, common under the POINT_SPECTRUM folder, contains target spectra files for all the targets (T1-T5). The folder envi_sli contains target spectra in ENVI file format named as T1, T2, T3, T4, and T5, while the ascii folder contains the target spectra in the ASCII file format and are designated as T1.txt, T2.txt, T3.txt, T4.txt and T5.txt. The folders: envi_sli and ascii, common under the PIXEL_SPECTRUM folder, contains target spectra files for targets T1, T2, T4, and T5. The other file conventions and details are similar, as described above. All the target spectra in the PROCESSED_SPECTRUM folder contains spectra in the range 0–1 and wavelength units of *nm*. The folder RAW_SPECTRUM contains the two subfolder – POINT_SPECTRUM and PIXEL_SPECTRUM, which contains the raw spectrum in point and pixel mode. The POINT_SPECTRUM subfolder under RAW_SPECTRUM contains the target spectra for all the targets (T1-T5) in SVC file format (T1.sig, T2.sig, T3.sig, T4.sig, and T5.sig), ENVI file format (T1.sli, T2.sli, T3.sli, T4.sli, and T5.sli with corresponding header (.hdr) files), and ASCII file(T1.txt, T2.txt, T3.txt, T4.txt, and T5.txt) format in the folders svc, envi_sli, and ascii respectively. The PIXEL_SPECTRUM subfolder under RAW_SPECTRUM contains the target spectra for the targets T1, T2, T4, and T5 in ENVI file format (T1, T2, T4, and T5 with corresponding header (.hdr) files), and ASCII file format (T1.txt, T2.txt, T4.txt, T5.txt) in the folders envi_sli, and ascii respectively.

The target's region of interest (ROI) files are organized in the folder TARGET_ROI (Fig. 1(c)). A 16 pixel ROI for airborne (AVNG folder) and 4 pixel ROI (T1, T2, T4, T5) and 6 pixels (T3) ROI for space-borne (SENT folder) is designated as the ground position of the targets. All the ROI files are the actual ground positions of the target recorded using a GPS device and contains the latitude and longitude of footprints of the targets. Folders T1, T2, T3, T4, and T5 contains the respective ROI files for both airborne as well as the space-borne sensor. A file naming convention: target_sensor_mode.extension is adapted for naming the respective ROI files. E.g., T1_A.shp describes the target ROI for the T1 target in the airborne mode available as .shp (shapefile). The ROI files are provided in .shp, .roi, and ASCII file format. The user must note that the .shp file is associated with .shx and .dbf file and are necessary for the .shp file to function.

## 2 Experimental Design, Materials and Methods

### 2.1 Data acquisition design

**Fig. 2:**
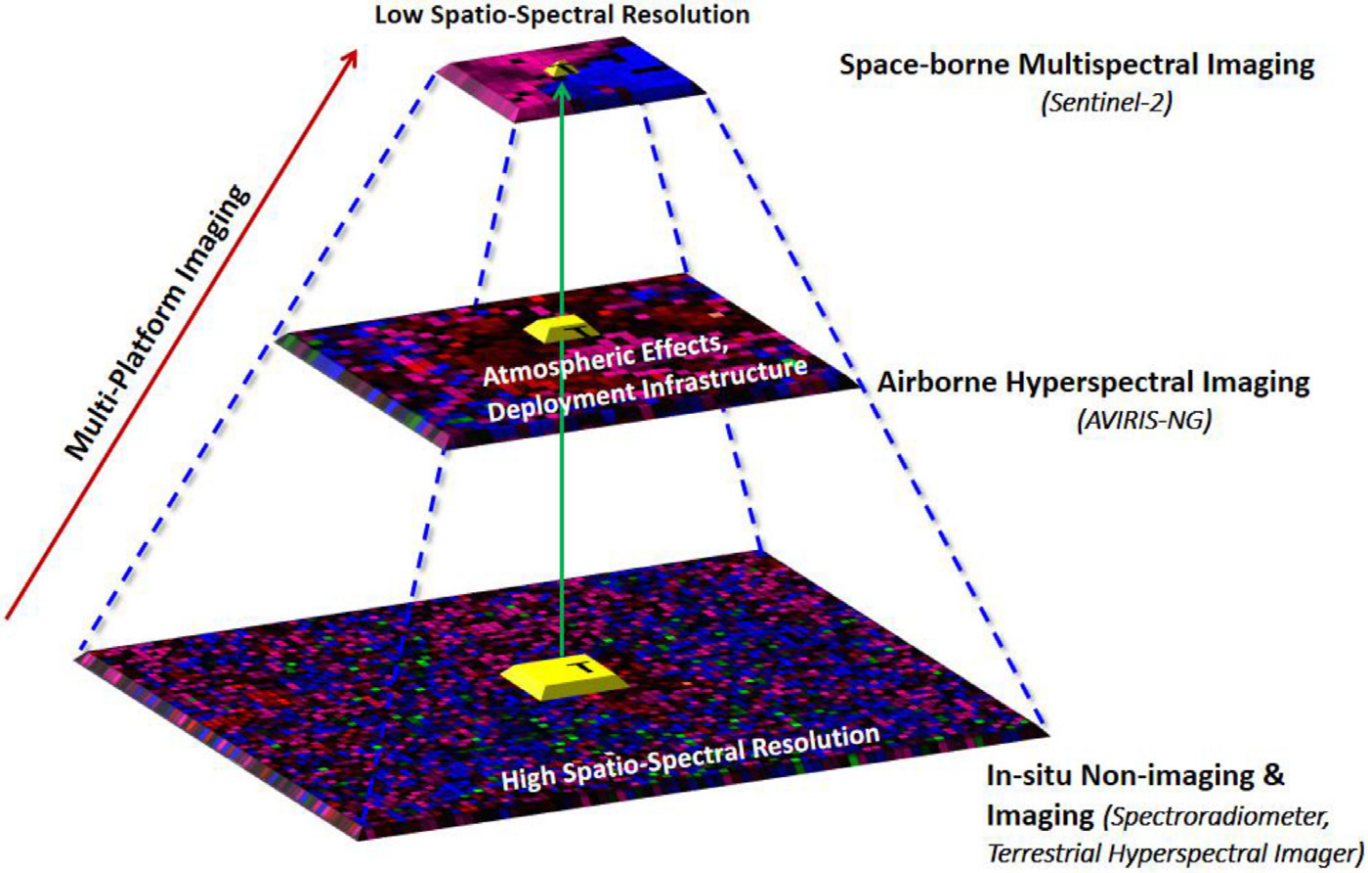
Multi-platform target detection data acquisition framework.

The conceptual design of the target detection dataset acquisition is as shown in Fig. 2. The experiment was conducted to explore the characteristic of detection dynamics ranging from ground to space-borne remote sensing platforms. Targets are positioned such that they are concurrently imaged from an ultra-high resolution (∼cm) ground-based imager to moderate resolution airborne and coarse resolution space-borne sensor. The proposed dataset was acquired on 20th March 2018 at Gudalur city, Tamil Nadu, India.

### 2.2 Targets

In this experiment, we have deployed five targets of different artificial thin-sheet materials of different colours (base material: nylon and cotton), each of the size 10 m x 10 m. For further details on target ground locations, readers can refer to the related research article [1]. The data deposited at http://dx.doi.org/10.17632/5ph8ms8p5n.2 contains the data for the targets specified as T1 (green nylon sheet), T2 (red nylon sheet), T3 (white cotton sheet), T4 (yellow nylon sheet), and T5 (black nylon sheet). We placed Targets T1, T2, and T3 on natural grass, and T4 and T5 on reflective soil backgrounds. We chose grass and soil backgrounds for T1 and T4 to introduce a camouflage in the visible spectral range of the electromagnetic spectrum. We used identical target materials (T1, T2, T4, and T5) with different colours, which could be a potential source to study the detectability of materials with broadly similar spectral reflectance characteristics. The ground position of the targets was recorded using a GPS device. A 16-pixel region of interest (ROI) and two ROIs of 4 and 6 pixels for space-borne imagery were designated as targets’ footprints [2]. Due to different imaging geometry and spatial resolutions of airborne and space-borne imagery, the ROI in space-borne imagery also contains sub-pixel targets.

### 2.3 Sensors

**Fig. 3:**
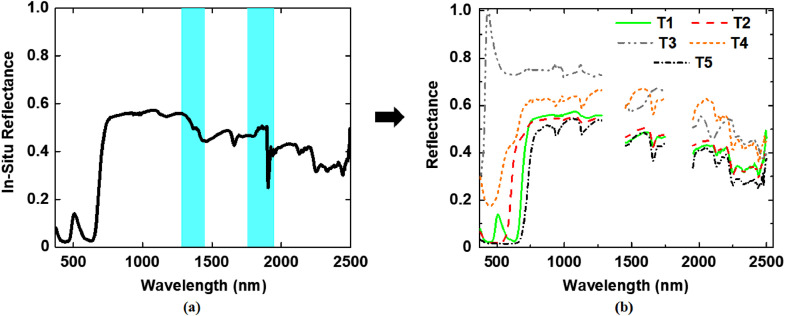
(a) Example of a raw in-situ reflectance spectrum of a target, and (b) processed reflectance spectra of targets.

We used the ground-based push-broom terrestrial hyperspectral imager (THI) (Headwall Photonics Inc., USA), airborne hyperspectral imager (AVIRIS-NG [3]), and the space-borne multi-spectral sensor, Sentinel-2 [4]. The THI, which captures hyperspectral imagery in the VNIR region (40–1000 nm) at about 1 nm spectral resolution, was mounted on a movable tripod-based platform and with sensor-target distance adjusted to image at a spatial resolution of about 1 cm. As part of the NASA and ISRO research collaboration for the HySI hyperspectral satellite [5], the airborne hyperspectral imagery were acquired over the targets at 4 m spatial resolution and 5 nm spectral resolution in the 400–2500 nm spectral range. The Sentinel-2 satellite acquires multi-spectral imagery at different spatial resolutions. We used the imagery acquired at 10 m and 20 m resolution corresponding to blue (490 nm), green (560 nm), red (665 nm), NIR (842 nm), and vegetation red edge (705 nm, 740 nm, 783 nm, 865 nm), SWIR(1610 nm, 2190 nm) bands for the proposed experimental dataset. We collected point-based in-situ hyperspectral reflectance measurements of the targets (Fig. 3) using a field spectroradiometer (Spectra Vista Corporation, HR-1024i, USA) which samples signals at 1.5 nm.

### 2.4 Data pre-processing

Atmospheric correction of the AVIRIS-NG hyperspectral imagery and Sentinel-2 satellite imagery was carried out using the Fast Line-of-sight Atmospheric Analysis of Spectral Hypercubes (FLAASH) model [6]. The spectral channels corresponding to the water vapour absorption regions of the electromagnetic spectrum, between 1348 and 1443 nm, 1804–1954 nm, and 2485–2500 nm, were removed from the data, thus resulting in effective imagery with 370 spectral bands. The Sentinel-2 imagery acquired at 20 m spatial resolution was resampled using the nearest neighbourhood algorithm to match the size of the targets. The in-situ target spectra were collected using a spectroradiometer as per the standard procedure [7]. The high-resolution THI derived imagery was used for generating the pixel-based in-situ target spectral reference by sampling the target pixels corresponding to different positions on the target materials. To avoid the effects of sensor noise beyond 900 nm in the THI imagery, we used the THI imagery acquired in the spectral range 400 nm to 900 nm. After the initial pre-processing, all the in-situ target reference spectra were spectrally resampled to conform to the spectral range and bandwidth of the of the respective sensor as described in [8].

## Declaration of Competing Interest

The authors declare that they have no known competing financial interests or personal relationships which have, or could be perceived to have, influenced the work reported in this article.
